# Management of Benign Prostatic Hyperplasia: Could Dietary Polyphenols Be an Alternative to Existing Therapies?

**DOI:** 10.3389/fphar.2017.00234

**Published:** 2017-04-28

**Authors:** Chinedum Eleazu, Kate Eleazu, Winner Kalu

**Affiliations:** ^1^Department of Chemistry/Biochemistry, Federal University Ndufu-Alike, IkwoAbakaliki, Nigeria; ^2^Department of Biochemistry, Ebonyi State UniversityAbakaliki, Nigeria; ^3^Department of Biochemistry, Michael Okpara University of AgricultureUmudike, Nigeria

**Keywords:** benign prostatic hyperplasia, polyphenols, nutrition, antioxidants, nutraceuticals

## Abstract

The incidence of benign prostatic hyperplasia (BPH) is gradually on the increase. While conventional drugs such as the α1-adrenergic receptor antagonists and 5α-reductase inhibitors have been found to be useful in the treatment of BPH, the adverse side effects associated with their usage, have led to increased search for alternative means of managing this disease. Furthermore, although surgery has also been suggested to be a sure method, the cost and risks associated with it excludes it as a routine treatment. Dietary polyphenols have gained public interest in recent times due to their roles in the prevention of various diseases that implicate free radicals/reactive oxygen species. However, their roles in the management of BPH have not been explored. Hence, this review on their prospects in the management of BPH and their mechanisms of action. Literature search was carried out in several electronic data bases such as PubMed, Google Scholar, Medline, Agora, and Hinari from1970 to 2017 to identify the current status of knowledge on this concept. The findings from these data bases suggest that while dietary polyphenols may not replace the need for the existing therapies in the management of BPH, they hold promise in BPH management which could be explored by researchers working in this field.

## Introduction

Benign prostatic hyperplasia (BPH) is the most common urological condition among elderly men, affecting approximately half of men over 80 years of age. It usually begins as a simple micronodular hyperplasia with a subsequent macroscopic nodular enlargement that may result in bladder outlet obstruction and the development of lower urinary tract symptoms (LUTS) (Aleksandra et al., 2015).

As the prostate enlarges, it constricts the urethra, inducing various symptoms such as: weak urinary stream, incomplete bladder emptying, nocturia, dysuria, and bladder outlet obstruction (In et al., 2012). These symptoms which have been associated with BPH are known as LUTS (Barkin, 2011).

Although the molecular and stromal mechanisms associated with the pathogenesis of this metabolic disorder have not yet been fully elucidated (Aleksandra et al., 2015), several factors such as: inflammatory mediators, hormonal, dietary factors, environmental and oxidative stress have been implicated in its etiology (Minciullo et al., 2015).

Currently, the two major medications that are being used for the treatment of BPH include the α1-adrenergic receptor antagonists (doxazosin, terazosin, and tamsulosin) which alleviate LUTS by relaxation of smooth muscle in the prostate and the neck of the bladder; and the 5α-reductase inhibitors [which inhibit the development of BPH through a reduction in dihydrotestosterone (DHT) production].

While these conventional drugs have been found to be efficacious in the treatment of BPH, the adverse effects associated with them, ranging from impotence and gynaecomastia to orthostatic hypotension, abnormal ejaculation amongst others, have led to increased search for alternative means of managing this disease.

Although surgery has also been suggested to be an option, the cost and risks associated with it excludes it as a routine treatment. The trend then is increased search for alternative methods of managing this disease using natural remedies (Kalu W. et al., 2016; Kalu W.O. et al., 2016).

Polyphenols are the most abundant dietary antioxidants and are common constituents of many plant food sources, including fruits, vegetables, seeds, nuts, chocolate, wine, coffee, and tea. Natural polyphenols have increasingly gained public interest in recent times. This could be attributed to the emerging evidence that suggests their roles in the prevention of various disease that implicate free radicals/reactive oxygen species (ROS; Scalbert et al., 2005; Eleazu et al., 2013a,b, 2017). However, the role of these polyphenols in the management of BPH and their mechanisms of action are yet to be reported. Hence, this article which reviewed the concept of management of BPH using dietary polyphenols.

## Materials and Methods

Literature search was carried out in several electronic data bases such as PubMed, Google Scholar, Medline, Agora, and Hinari from1970 to 2017 to identify the current status of knowledge on the contribution of dietary polyphenols to the management of BPH and their mechanisms of action. The findings from these data bases are therefore reported in this review.

### Definition of Polyphenols

Polyphenol is a generic term for thousands of plant-based molecules that possess antioxidant properties. These polyphenols have been identified as secondary metabolites of plants that contain one or more hydroxyl groups attached to the ortho, meta or para positions on a benzene ring (Rajasekaran et al., 2001).

Over 8,000 identified polyphenols are present in foods and they have been found to play important roles in the maintenance of human health and wellness (Tsao, 2010; Eleazu et al., 2013b; Eleazu, 2016).

### Classification of Polyphenols and Their Dietary Sources

Polyphenols could be classified into five groups namely: (a) phenolic acids, (b) stilbenes, (c) lignans (d) flavonoids, and (e) curcuminoids (Eleazu and Eleazu, 2015; Ly et al., 2015; Eleazu et al., 2017).

(a) Phenolic acids are further divided into two subgroups which are: (i) hydroxybenzoic acids (example is gallic acid), found in tea and (ii) hydroxycinnamic acids. Examples of hydroxycinnamic acids are caffeic acid found in virtually all fruits (Manach and Donovan, 2004); chlorogenic acid found in strawberries, pineapple, etc; and *p*-coumaric acid that is found in cereal grains (Ly et al., 2015).
(b) Stilbenes are found in red wines, red grape juice and peanuts (Vitrac et al., 2002; Prasad, 2012) while resveratrol is the most common.
(c) Lignans: These groups of polyphenols include: secoisolariciresinol found in flaxseed (Ly et al., 2015) and sesamin found in sesame seed (Smeds et al., 2012).
(d) Flavonoids could be found in fruits, vegetables, legumes, red wine, and green tea. They are further divided into eight subgroups namely: flavanols, flavonols, flavones, isoflavones, flavanones, anthocyanins, proanthocyanidins, and chalcones.
  Flavanols (example is epigallocatechin gallate) are found in green and black tea (Khan and Mukhtar, 2007).
  Flavonols (examples are kaempferol and quercetin) could be found in onions, broccoli, and blueberries (Hollman and Arts, 2000).
  Anthocyanins (example is cyanin glucoside) could be found in highly pigmented fruits (Ly et al., 2015).
  Flavones (examples include: apigenin, chrysin, and luteolin) could be found in Parsley and Celery (Ly et al., 2015).
  Isoflavones (examples are daidzein and genistein) are found in soya and its processed products (Cassidy et al., 2000).
  Flavanones (example is naringenin) could be found in grapefruit (Ly et al., 2015).
  Chalcones are mainly abundant in beer. They are also abundant in fruits like citruses and apples; in certain vegetables such as shallot, tomatoes, potatoes, and bean sprouts and in various plants and spices (licorice, cardamom). Xanthohumol is the main prenylated chalcone, predominately found in beer (Mojzer et al., 2016; Eleazu et al., 2017).
(e) Curcuminoids represent another class of polyphenols; a typical example being curcumin which is found in *Curcuma longa* (Eleazu et al., 2017).

### Metabolism, Absorption, and Bioavailability of Polyphenols

After metabolism of polyphenols by Phase I and II enzymes of xenobiotic metabolism, weakly conjugated polyphenols re-enter circulation, while extensively conjugated polyphenols are excreted in the bile and enter the large intestine. The microflora hydrolyzes glycosides into aglycones and then metabolizes the aglycones into different aromatic acids, which are well absorbed across the colonic barrier (Han et al., 2007; Knaup et al., 2007; Eleazu et al., 2017).

The physicochemical properties of polyphenols (such as: molecular weight, extent of glycosylation and esterification) determine their intestinal absorption (Eleazu et al., 2017). Polyphenols in the form of esters and glycosides are absorbed less rapidly and less efficiently than aglycones and glucosides (Manach and Donovan, 2004) due to the fact that glycosylated polyphenols are hydrophilic and thus are unable to passively diffuse through the gut wall until they are hydrolyzed (Nemeth et al., 2003; Mojzer et al., 2016). This provides an explanation for the low absorption of dietary polyphenols in the stomach as most of them are mostly present in glycosylated forms with one or more sugar residues conjugated to a hydroxyl group or the aromatic ring.

While the actual bioavailability of dietary polyphenols is yet to be fully understood, there are indications that the prostate gland is one of the tissues that readily incorporate them (Eleazu et al., 2017). For instance, studies carried out by Abd et al. (2006) indicated that polyphenols were detected by HPLC technique in a number of tissues in mice and rats, one of which is the prostate, suggesting their bioavailability in the prostatic tissue. In another study (Henning et al., 2006) that investigated the bioavailability of tea polyphenols and theaflavins in human serum and human and mouse tissues, these polyphenols were found in the conjugated and free forms in the prostate tissue in addition to other tissues. These reports thus suggest the prostate gland to be one of the tissues where dietary polyphenols exert their biological actions.

## Etiology of BPH

Benign prostatic hyperplasia is a major health concern and which incidence is expected to increase in line with the greater life expectancy. A number of factors have been implicated in its etiology and which factors include: aging, hormonal alterations, metabolic syndrome, inflammation, oxidative stress (Roehrborn and McConnell, 2002), and more recently, suppression of apoptosis in the prostatic tissue.

### Aging and BPH

Aging has been implicated as the major risk factor for the development of BPH (Roehrborn and McConnell, 2002; Aleksandra et al., 2015). Several studies have demonstrated a relationship between age and markers of BPH progression (Neuhouser et al., 2008; Liu et al., 2009).

In aging males, tissue remodeling occurs within the prostate especially in the transition zone. The most significant modifications occur in the basal cells which change their intracellular metabolism leading to prostatic enlargement. The nodular enlargement is androgen dependent and the tissue remodeling involves both the epithelium and fibromuscular stroma (Kalu W. et al., 2016; Kalu W.O. et al., 2016).

### Hormonal Alteration and BPH

The growth and malignant transformation of the prostate gland have been reported to be influenced by sex hormone levels. Although androgens do not cause BPH, the development of BPH requires the presence of testicular androgens during prostate development, puberty, and aging (Kalu W.O. et al., 2016). Reports also have it that bioavailable prostatic testosterone levels decline with age (Alberto et al., 2009).

Luminal secretory cells require androgens, especially the intracellular metabolite of testosterone, DHT, for terminal differentiation and secretory functions. Testosterone is converted to DHT by the intracellular enzyme, 5α reductase type 2 (Roehrborn and McConnell, 2002; Alberto et al., 2009; Aleksandra et al., 2015) which is located on the prostatic nuclear membrane for both the stroma and the epithelium (Roehrborn and McConnell, 2002; Aleksandra et al., 2015).

Dihydrotestosterone can act in an autocrine fashion on the stromal cells or in paracrine fashion by diffusing into nearby epithelial cells. In both of these cell types, DHT binds to nuclear androgen receptors and signals the transcription of growth factors that are mitogenic to the epithelial and stromal cells leading to prostatic hyperplasia. DHT is ten times more potent than testosterone because it dissociates from the androgen receptor more slowly. The importance of DHT in causing nodular hyperplasia is supported by clinical observations in which finasteride, an inhibitor of 5α-reductase is given to men with BPH. Therapeutic use of the 5α-reductase inhibitors markedly reduces the DHT content of the prostate and, in turn, reduces prostate volume and BPH symptoms (Alberto et al., 2009).

There are indications that estrogens may also contribute to the etiology of BPH (Kalu W.O. et al., 2016). Reports have it that as men age, their testicular function decreases as are their androgen levels. Furthermore, the conversion of androgen to estrogen is increased (Zhou et al., 2010). In addition, while circulating levels of free estradiol remain constant in the aging man due to an age-related increase in body weight and adipose cells, the increase in the adipose cells leads to the expression of high levels of aromatase that produces estrogen conversion. The result is that the plasma estrogen to androgen ratio increases, resulting in decreased inhibition of androgens on estrogen release. The released estrogens cause increased stimulation of the prostatic stroma, resulting in excessive proliferation of the prostate and occurrence of BPH (Alberto et al., 2009; Ho and Habib, 2011; Kalu W.O. et al., 2016). Estrogen induced stimulation of prostatic growth has also been reported in dogs and monkeys (Jeyaraj et al., 2000; Roehrborn and McConnell, 2002; Aleksandra et al., 2015).

### BPH and Metabolic Syndrome

Metabolic syndrome is a well-recognized cluster of cardiovascular risk factors including obesity, hypertension, dyslipidemia, and hyperglycemia, closely associated with an increased risk of cardiovascular diseases (Corona et al., 2014).

Hammarsten and Högstedt (2001) in their studies reported that patients with hypertension, and obesity were at risk of developing BPH. Epidemiologic studies also provided evidence that hypertensive men are more likely to develop BPH and to undergo medical and surgical therapy than healthy men (McVary, 2006). These studies hypothesized that the noradrenergic nerves may contribute to the functional component of the bladder outlet obstruction due to BPH.

Recent studies (Aleksandra et al., 2015) also confirmed the frequent coexistence of metabolic syndrome and BPH. This association was suggested by the authors to be a result of the metabolic syndrome-related metabolic derangements, changes in the sex-hormone and lowered sex-hormone binding protein levels.

### BPH and Oxidative Stress

Oxidative stress refers to an imbalance between the production and detoxification of ROS/free radicals in favor of ROS/free radicals that can cause tissue damage (Eleazu et al., 2013b, 2017; Minciullo et al., 2015).

Reactive nitrogen species (RNS) and ROS are by-products of normal cellular metabolism which impact on cell signaling. Increases in the levels of these ROS and RNS induce oxidative stress (Udensi and Paul, 2016) by causing a significant decrease in antioxidant defense mechanisms, leading to protein, lipid and DNA damage and subsequent disruption of cellular functions and cell death.

Oxidative stress has been considered to be one of the mechanisms that trigger the chain of reactions involved in the development and progression of prostatic hyperplasia. This is especially true as the human prostate tissue is vulnerable to oxidative DNA damage due to more rapid cell turnover and fewer DNA repair enzymes (Minciullo et al., 2015).

Recent studies carried out by Udensi and Paul (2016), showed the levels of antioxidants in the prostatic tissue to be significantly decreased in prostatic hyperplasia (Udensi and Paul, 2016). Furthermore, our study (Kalu W. et al., 2016) on animal models of BPH showed significant elevation of prostatic lipid peroxidation with concomitant significant reduction of the prostatic levels of GSH, SOD, GPx, and catalase activities of BPH untreated rats and which parameters were significantly improved following treatment with finasteride or kolaviron.

### BPH and Inflammation

Inflammation is a natural defense mechanism against pathogens and it is associated with many pathogenic diseases such as microbial and viral infections, exposure to allergens, radiation and toxic chemicals, autoimmune and chronic diseases, obesity, consumption of alcohol, tobacco use, etc. (Tarique et al., 2016).

Previous studies on the pathogenesis of BPH indicated the differential expression of cytokines in BPH tissue suggesting a role of inflammation in the development of BPH (Lee and Peehl, 2004). Clinical studies carried out also support the role of inflammation in BPH. For instance, Di Silverio et al. (2003) when analyzing the clinical and pathologic data of approximately 4000 patients who had undergone transurethral resection of the prostate or open prostatectomy for BPH, found evidence of acute or chronic inflammation in 42.5% of the patients. Their studies showed a statistical significant correlation between prostate size and both acute and chronic inflammation, with neuthrophic or mononuclear infiltrates present in 29.9, 37.3, and 50% of the prostates, respectively. Other reports also correlated the degree of inflammation in patients with BPH with the prostatic size (Mishra et al., 2007; Nickel et al., 2008; Hamid et al., 2011).

Inflammation is also one of such ways of generating free radicals/ROS in the prostatic tissue. Prostatic inflammation can cause the generation of free radicals/oxygen species such as inducible nitric oxide synthase (iNOS), reactive nitric species and ROS (Hamid et al., 2011). For instance, while iNOS is not detected in normal prostate, it is expressed in the prostate of all BPH patients (Minciullo et al., 2015).

Prostaglandins represent a group of inflammatory mediators that result from the conversion of arachidonic acid mediated by two different isoforms of cyclooxygenase (COX) (COX-1 and COX-2), both of which forms are expressed in the prostate gland (Altavilla et al., 2012).

Cyclooxygenase-2 is a pro-inflammatory, inducible enzyme whose production is triggered by mitogens, cytokines, ROS, and growth factors in different cell types. Increased expression of COX-2 mRNA has been reported in BPH (Kirschenbaum et al., 2000; Altavilla et al., 2012). Several mechanisms have been proposed to explain the role of COX-2 in prostatic hyperplasia and one of which mechanisms involves COX-2- mediated increases in prostaglandin synthesis, especially of prostaglandin E2.

In rodent models, induced prostatic inflammation was also shown to contribute to prostatic hyperplasia (Kessler et al., 1998; Hamid et al., 2011). These studies amongst others (Kramer et al., 2007; Sciarra et al., 2008; Kroeger et al., 2013) are pointers to the contribution of inflammation to the development of BPH.

### BPH and Apoptosis

Apoptosis plays an important role in the control of cell growth and in the maintenance of tissue homeostasis (Minutoli et al., 2014).

There are indications that the apoptosis machinery could be a promising therapeutic target for BPH. This is because cell growth arises from either increased cell proliferation or decreased cell death (Alberto et al., 2009) and BPH has been shown to result from smooth muscle and epithelial proliferation primarily within the prostatic transition zone that can produce LUTS (Minutoli et al., 2014).

After physiologic growth to adult size, the prostate enters a maintenance phase, where prostatic cell proliferation occurs at a daily rate of 1–2% counter balanced by an equal rate of programmed cell death (Shariat et al., 2005). A lack of this balancing has been implicated in the development of BPH (Kyprianou, 2003; Giacomo et al., 2006).

Several studies suggested that a reduction of apoptosis might occur in BPH. For instance, the study carried out by Kyprianou et al. (1996) showed that basal and luminal epithelial cells in BPH over-express Bcl-2 (apoptotic suppressor), compared to healthy prostate tissue.

Another study also showed that survivin, an inhibitor of apoptosis that counteracts cell death and controls mitotic progression was over expressed in the stromal compartment of patients with BPH (Shariat et al., 2005).

Investigation into the mechanisms of action of the drugs that are currently being used in BPH management suggested possible interactions with apoptotic mechanisms (Giacomo et al., 2006). For example, previous studies on rat prostates showed that finasteride induced apoptosis in epithelial cells, inhibiting insulin-like growth factors (IGF) I and 1R expressions (Huynh et al., 1998). These authors also showed that finasteride down-regulated the expression of the anti-apoptotic proteins- Bcl-xL and Bcl-2, making the prostate cells more susceptible to apoptosis. Other studies on BPH patients treated with finasteride further affirmed the pro-apoptotic effect of finasteride on the epithelial cells (Saez et al., 1998), and increased expression of the pro-apoptotic proteins (Caspases-3, -6, and -9) during finasteride treatment of BPH (Aline et al., 2005). Recent studies also showed increased expression of inhibitors of apoptosis proteins in human prostates with BPH (Rodriguez-Berriguete et al., 2010; Minutoli et al., 2014).

## Dietary Polyphenols and Inflammation

Inflammation is an immunological defense mechanism by which tissues respond to an insult (Ly et al., 2015). A number of polyphenols such as resveratrol, curcumin, catechins, and others have been extensively investigated as anti-inflammatory agents (Hatcher et al., 2008). In addition, anti-inflammatory activities of other polyphenols such as quercetin, rutin, morin, hesperetin, and hesperidin have been reported in acute and chronic inflammation in animals (Aruna et al., 2014; Yang and Lim, 2014; Tarique et al., 2016).

Polyphenols have been reported to exert their anti-inflammatory properties at multiple levels, through the modulation of mitogen-activated protein kinases (MAPK) signaling pathways and NF-κB and AP-1 transcription factors, inhibition of the production of inflammatory cytokines and chemokines, suppressing the activity of COX-2 and iNOS (Donnelly et al., 2004), thereby decreasing the production of ROS/RNS (Ichikawa et al., 2004; Pandey and Rizvi, 2009).

## Dietary Polyphenols and Oxidative Stress

Oxidative stress occurs when there is an imbalance between the pro-oxidants and the antioxidants in a cell system in favor of the pro-oxidants which can result in damage to DNA, proteins and lipids as earlier defined.

Consumption of polyphenol rich foods, fruits, vegetables, and beverages is considered of immense benefit to human health (Vasundev, 2006) as these polyphenols are able to directly scavenge free radicals and inhibit metal-mediated free radical formation (Frei and Higdon, 2003). When compared with endogenous antioxidants, the importance of dietary polyphenols/antioxidants *in vivo* as oxidant scavengers is considered to be minor due to their lower reduction potentials and bioavailability (Frei and Higdon, 2003). However, these dietary polyphenols have been shown to play greater roles in the prevention of oxidative stress through mechanisms such as: (i) inhibition of redox-sensitive transcription factors [example is nuclear factor-kB (NF-kB)] (ii) down-regulation of pro-oxidant enzymes (example is iNOS and COX-2) and (iii) induction of Phase II enzymes (Frei and Higdon, 2003; Siddiqui et al., 2008; Eleazu et al., 2017).

In recent times, emerging evidence has indicated that apart from the antioxidant activities of polyphenols, polyphenols can also directly or indirectly strengthen the degradation of misfolded and damaged proteins.

Under normal physiological conditions, proteins undergo different conformational changes during their lifetime. Each step is assisted by chaperones and includes *de novo* folding, assembly and disassembly, transport across membranes, and targeting for degradation (Hajieva, 2017). Therefore, in optimal conditions, long-term health is maintained by protein homeostasis which is a complex network of molecular interactions that balances protein biosynthesis, folding, translocation, assembly, disassembly, and clearance (Dattilo et al., 2015).

The expression of many chaperones is strongly induced under conditions of oxidative stress and heat shock challenges. Therefore, they are considered to be stress proteins or heat shock proteins (Hajieva, 2017) and the ability of a cell to counteract stressful conditions is referred to as cellular stress response or heat shock response (Dattilo et al., 2015).

Production of heat shock proteins, including protein chaperones has been reported to be important for the folding and repair of damaged proteins, leading to cell survival conditions that would have led to apoptosis (Dattilo et al., 2015).

Recently, it has been shown that polyphenols can also directly or indirectly strengthen the degradation of misfolded and damaged proteins as earlier stated. This can be achieved by increasing the activity and efficiency of the cellular protein degradation machinery (Chhangani and Mishra, 2013; Hajieva, 2017). Pharmacologically active polyphenols and their derivatives have been reported to play significant roles in stabilizing the protein degradation machinery at different stages which may be an attractive therapeutic strategy to halt the accumulation of misfolded proteins (Hajieva, 2017). For instance, resveratrol 20 was shown to induce the acute heat shock response by upregulating chaperones like Hsp70 (Putics et al., 2008). Curcumin 18 was reported to demonstrate cytoprotective effects and induce nuclear translocation of HSF1 (Teiten et al., 2009) while also demonstrating anti-inflammatory and antioxidant activities (Hajieva, 2017).

Studies by Scapagnini et al. (2011) showed that curcumin induces hemeoxygenase-1 (HO-1, a heat shock protein) expression and activity in different brain cells via the activation of heterodimers of NF-E2-related factors 2 (Nrf2)/antioxidant responsive element pathway. Furthermore, activation of Nrf2 target genes, and particularly HO-1, in astrocytes and neurons is strongly protective against oxidative damage and cell death (Scapagnini et al., 2011).

In another report, Fetoni et al. (2015) showed that the polyphenol-rosamaric acid attenuated noise-induced hearing loss (which is associated with increase of ROS and lipoperoxidative damage together with the imbalance of antioxidant defenses), through activation of the redox-sensitive transcription factor nuclear factor erythroid 2-related factor 2/HO-1 pathway.

## Preclinical Studies Reporting on Management of BPH with Dietary Polyphenols and their Mechanisms of Action

Currently there are dietary polyphenols that have demonstrated 5α-reductase inhibitory properties which makes them promising agents for the management of BPH, but which activities would need to be confirmed using *in vivo* models of BPH/clinical studies. For instance, the reports of Hiipakka et al. (2002) working on cell free assays and intact cells, showed that the dietary polyphenols- myricetin, baicalein (5,6,7-trihydroxyflavone), fisetin, daidzein, kaempferol, caffeic acid phenethyl ester, octyl and dodecyl gallates inhibited human 5α-reductase activities. While *in vivo*/clinical studies will be needed to confirm these studies, these authors speculated that since these polyphenols were effective on whole cells, they could be capable of modulating the activity of 5α-reductase *in vivo*. Studies conducted by Park et al. (2006) also confirmed the 5α-reductase inhibitory activity of kaempferol and caffeic acid phenethyl ester as previously reported by Hiipakka et al. (2002).

On the other hand, there are dietary polyphenols that were reported in preclinical studies to possess the potentials of suppressing BPH. Typical examples are the isoflavonoids- genistein, biochanin A and equol and the lignan- enterolactone which were reported to ameliorate BPH in BPH tissue homogenates through inhibition of 5α-reductase activity (Evans et al., 1995).

ACTICOA powder (contains mostly flavonols), a cocoa polyphenol extract was demonstrated by Bisson et al. (2007) to be useful in ameliorating testosterone propionate-induced prostate hyperplasia in rats.

Again, soybean isoflavones were reported to inhibit BPH and increase the expressions of nitric oxide and nitric oxide synthase (Yang et al., 2009).

Similarly, quercetin (from onions, red wine, tea, cranberry, and citrus fruits) was reported to suppress BPH in preclinical studies (Tamler and Mechanick, 2007; Borovskaya et al., 2015) although the mechanism of action is yet to be ascertained.

In another report, flavocoxid, a flavonoid from *Scutellaria baicalensis* (Chinese skullcap) and *Acacia catechu* (black catechu), respectively, was reported to demonstrate anti-BPH properties and the mechanism of action was reported to involve decreasing growth factor expression and inhibition of COX-2 and 5-lipoxygenase activities in the prostatic tissue of BPH rats (Altavilla et al., 2012).

Equol (4′,7-isoflavandiol), a polyphenol/isoflavonoid molecule derived from soya and intestinal metabolism (Axelson et al., 1984), was reported to suppress BPH in experimental studies. According to the authors, this polyphenol has potent antioxidant and anti-aging properties to decrease prostatic irritation and potentially neoplastic growth. The mechanism of its ameliorating action on BPH was reported as its specific binding to 5α-DHT by sequestering 5α-DHT from the androgen receptor, thus decreasing androgen hormone actions to improve prostate health by acting as a selective androgen modulator (Edwin, 2014).

Lycopene, a polyphenol commonly found in tomato, was shown to decrease BPH in animal models of BPH. The mechanism of action was reported to be stimulation of increased expression of the pro-apoptotic protein- caspase-3 and suppression of inflammatory marker- IL-6 (Minutoli et al., 2014).

In another study (Hoon et al., 2010), anthocyanins from black soybean were reported to suppress prostatic hyperplasia in rats induced with BPH. The mechanism of action was reported by the authors to involve suppression of cellular proliferation through induction of apoptosis in the prostatic tissue.

In a more recent study using animal models, we showed the beneficial role of kolaviron, a polyphenol and a biflavonoid complex from *Garcinia kola* in the management of BPH in BPH challenged rats (Kalu W. et al., 2016; Kalu W.O. et al., 2016). The mechanism of action was found to involve inhibition of 5-alpha reductase and oxidative stress in the prostatic tissue. Furthermore, Jinglou and Hongping (2016) in their recent study reported that epigallocatechin-3-gallate had protective effects against testosterone induced BPH in rats and against BPH in metabolic syndrome rats. The mechanism of the BPH protective action of this polyphenol on the experimental BPH induced rats was reported by the authors to involve repair of the antioxidant defense mechanisms (superoxide dismutase and glutathione peroxidase) in the prostate with concomitant diminution of prostatic levels of malonaldehyde; diminution of the prostatic levels of the inflammatory markers- interleukin (IL)*-*Iβ, IL-I6, tumor necrosis factor-α (TNF*-*α); inhibition of IGF-I and IGF-II as well as upregulation of peroxisome proliferator-activated receptors (PRAR) – α and PPAR-γ that have been shown to contribute to apoptosis. Another very recent study (Ren et al., 2016) also reported the treatment effects of flaxseed-derived secoisolariciresinol diglycoside (a lignan extracted from flaxseed) and its metabolite enterolactone on BPH. The mechanistic basis for its anti-BPH activity was reported by the authors to involve stimulation of increased expression of G-protein-coupled estrogen receptor 1 (GPER) that has been reported to decrease cell proliferation by activating apoptosis in some cells (Chimento et al., 2013).

## Clinical Studies/Management of BPH with Dietary Polyphenols

Until now, the concept of management of BPH using dietary polyphenols has been under debate due to a number of clinical studies that gave contrary results as well as the adverse effects some of these polyphenols could pose following long term exposure.

For instance, in the review by Curtis et al. (2008), they listed some phytotherapies that are used in BPH management such as quercetin and others but showed the lack of clinical effects of some of these phytotherapies although clinical study conducted on BPH management with quercetin (Shoskes et al., 1999) and reviewed by Curtis et al. (2008), did not deny the anti-BPH effect of quercetin. Moreover, the double-blind randomized clinical trial conducted by Ghorbanibirgani (2012) provided evidence of the efficacy of quercetin in the treatment of BPH.

Again, clinical studies conducted on BPH management with resveratrol as reported by Kjaer et al. (2015) did not support the management of BPH with this polyphenol.

However, some other clinical studies carried out on BPH management with some dietary polyphenols provided evidence of their usefulness in the management of BPH.

For example, in a study carried out on the effects of a low oral dose of equol supplement (6 mg, twice a day with meals) for 4 weeks in a total of 18 men (49–60 years old) with moderate or severe BPH (Lephart, 2013), it was shown that a low dose of equol positively improved moderate to severe BPH symptoms according to the IPSS indices. In moderately symptomatic men, 5 out of 7 of the IPSS parameters significantly improved by 4 weeks of equol treatment while in severely symptomatic men, all 7 of the IPSS parameters significantly improved with 4 weeks of equol treatment. In addition, their study also showed that 5α-DHT levels declined by 21% in severely symptomatic men.

In a randomized, double-blind, placebo-controlled trial that investigated the effects of lycopene supplementation in elderly men diagnosed with BPH (Schwarz et al., 2008), it was reported that lycopene inhibited BPH progression and ameliorated the BPH symptoms in the patients at a dose of 15 mg/day for 6 months and which finding was supported by later experimental studies conducted by Minutoli et al. (2014) on the management of BPH with lycopene. Furthermore, the authors (Schwarz et al., 2008) also reported the lycopene supplements to be safe and well tolerated.

Similarly, in a 4 months randomized, double-blind, placebo-controlled clinical trial carried out in 87 subjects with BPH receiving treatment dosages of 0 (placebo), 300, or 600 mg/day of the flaxseed lignan extract (secoisolariciresinol diglucoside (SDG) (Zhang et al., 2008), the authors showed that for the 0, 300, and 600 mg/day SDG groups, respectively, the International Prostate Symptom Score decreased by -3.67 ± 1.56, -7.33 ± 1.18, and -6.88 ± 1.43 (mean ± SE, *P* = 0.100, <0.001, and <0.001 compared to baseline), while the Quality of Life score improved by -0.71 ± 0.23, -1.48 ± 0.24, and -1.75 ± 0.25 (mean ± SE, *P* = 0.163 and 0.012 compared to placebo. In addition, the authors suggested the therapeutic efficacy of this polyphenol to be comparable to that of the commonly used intervention agents of alpha1A-adrenoceptor blockers and 5α-reductase inhibitors. The finding by these authors with respect to BPH management using this polyphenol was further affirmed by the later experimental study conducted by Ren et al. (2016).

Similarly, the double-blind randomized clinical trial conducted by Ghorbanibirgani (2012) provided evidence of the efficacy of quercetin in the treatment of BPH as earlier stated.

Other clinical studies carried out supported the use of phytotherapies from different plants such as Eviprostat (Matsumoto et al., 2010), *Pygeum africanum* extract (Chatelain et al., 1999), Stinging nettle root extract (Bazotonuno) (Schneider and Rübben, 2004), PR-2000 (*Tribulus terrestris*) (Sahu and Kumar, 2001), and others in the management of BPH but which are beyond the scope of this review as the actual polyphenols in these phytotherapies were not indicated in these studies.

## Adverse Effects Associated with Uncontrolled and Long-Lasting Supplementation with Dietary Polyphenols

While the contribution of some dietary polyphenols in the management of BPH has been indicated in this review as supported by clinical findings, it must also be emphasized that non-regulated and long-lasting supplementation with some dietary polyphenols could lead to potential harmful effects due to possible interactions with cytochrome P450 (CYP) enzymes (Mancuso, 2015).

Amongst the CYP enzymes which are responsible for the metabolism of a wide array of endogenous substances, (example-steroids, hormones, lipids, and bile acids), xenobiotics (example-drugs and pollutants from the environment), and dietary products and/dietary polyphenols, CYP 3A4 has been shown to be the major enzyme that is involved in the metabolism of drugs and xenobiotics in the liver and gut (Loai and Zohar, 2015). It has also been shown to be expressed in the prostate, breast, gut, colon, small intestine, and the brain (Ferguson and Tyndale, 2011; Loai and Zohar, 2015).

In recent times, studies have also suggested possible interactions between dietary polyphenols and CYP 3A4, which could lead to some adverse effects.

For instance, Mancuso and Barone (2009) reported that curcumin and its derivatives inhibit the activity of CYP3A4 and other drug-metabolizing enzymes such as: glutathione-*S*-transferase and UDP-glucuronosyltransferase. These authors reported that inhibition by curcumin, alone or in combination with piperine, of CYP3A4 could be harmful, especially during prolonged usage.

Kimura et al. (2010) in their study, showed the inhibitory effects of polyphenols on human CYP3A4 and CYP2C9 activity *in vitro* which inhibitory actions were reported by the authors to involve the formation of a covalent bond between the polyphenol and the CYP3A4 molecule, leading to the inactivation of the enzyme, or reversible binding that causes reversible inhibition. Similarly, the-flavonols kaempferol, quercetin, and galangin were reported to inhibit CYP3A4-mediated metabolism of xenobiotics *in vitro* (Shimada et al., 2010; Loai and Zohar, 2015). Furthermore, inhibitory effects of catechins on CYP3A4 were reported in some studies (Kimura et al., 2010; Yang and Pan, 2012), although no specific mechanisms of action were reported in these studies.

One thing most of these studies reporting on the interaction of polyphenols with CYP enzymes have shown is the need for controlled/regulated use of polyphenols for therapeutic purposes.

**FIGURE 1:**
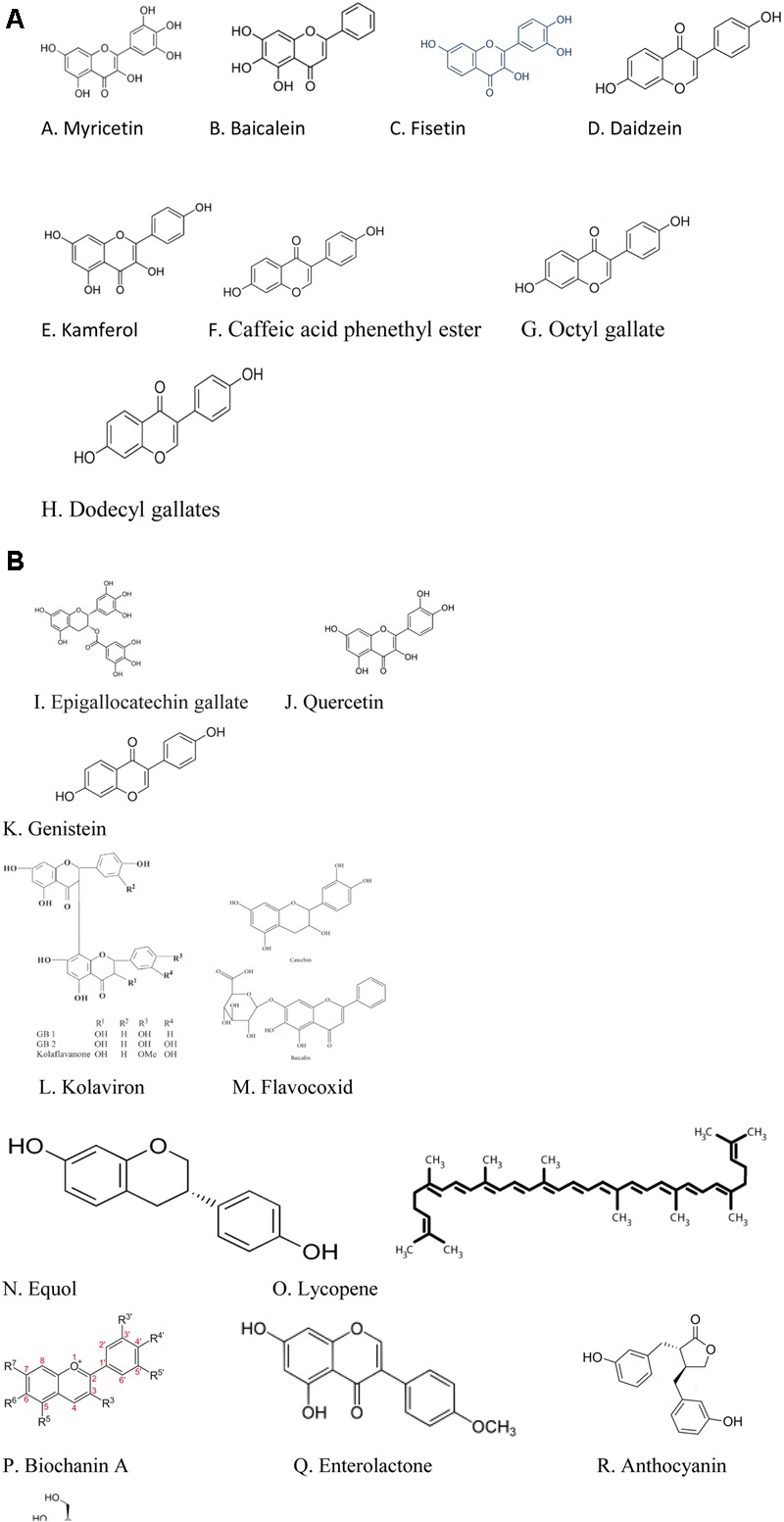
**(A)** Structures of the polyphenols with 5α-reductase inhibitory actions. **(B)** Structures of polyphenols with BPH suppressing actions in preclinical studies.

**Table 1** shows the dietary polyphenols identified in this review with 5α-reductase inhibitory activities but which activities would need to be confirmed using *in vivo*/clinical studies; **Table 2** summarizes the mechanisms through which the dietary polyphenols identified in the preclinical studies with BPH suppressing properties exert their biological effects; the structures of the polyphenols with 5α-reductase inhibitory properties are shown in **Figure 1A** while the structures of the polyphenols identified in the preclinical studies with BPH suppressing properties are shown in **Figure 1B**.

**Table 1:** Dietary polyphenols identified with 5α-reductase inhibitory actions.

Polyphenols	Source	Activity
Myricetin	Red wine	5α-reductase inhibition (Hiipakka et al., 2002)
Baicalein	*Scutellaria baicalensis* and *Scutellaria lateriflora*	5α-reductase inhibition (Hiipakka et al., 2002)
Fisetin	Strawberries, apples, grapes	5α-reductase inhibition (Hiipakka et al., 2002)
Daidzein	Soybeans	5α-reductase inhibition (Hiipakka et al., 2002)
Kaempferol	Apples, broccoli, onions, tomatoes	5α-reductase inhibition (Hiipakka et al., 2002; Park et al., 2006)
Caffeic acid-phenethyl ester	Propolis	5α-reductase inhibition (Hiipakka et al., 2002; Park et al., 2006)
Octyl gallate	Octanol and gallic acid (produced from plant tannins)	5α-reductase inhibition (Hiipakka et al., 2002)
Dodecyl gallates	Gallic acid (from plan tannins)	5α-reductase inhibition (Hiipakka et al., 2002)

**Table 2:** Dietary polyphenols identified with BPH suppressing properties.

Polyphenol	Source	Mechanisms of action
Epigallocatechin-gallate	Green tea	Suppression of oxidative stress, diminution of inflammatory markers (IL-Iβ, IL-I6, and TNF-α; inhibition of IGF-I and IGF-II and upregulation of PPAR-α and PPAR-γ (Jinglou and Hongping, 2016)
Lignan	Flaxseeds, sesame seeds	5α-reductase inhibition (Evans et al., 1995)
Genistein	Fava beans, soybeans	5α-reductase inhibition (Evans et al., 1995)
Biochanin A	Soy, Peanuts	5α-reductase inhibition (Evans et al., 1995)
Enterolactone	Flaxseed and sesame sees	5α-reductase inhibition (Evans et al., 1995)
Flavocoxid	*Scutellaria baicalensis* and *Acacia catechu*	Inhibition of growth factor expression and suppression of inflammation through inhibition of cyclooxygenase-2 and 5-lipoxygenase activities (Altavilla et al., 2012)
Equol	Soya	Binding to 5α-DHT by sequestering 5α-DHT from the androgen receptor (Evans et al., 1995; Edwin, 2014)
Anthocyanin	Soya	Suppression of cellular proliferation through induction of apoptosis (Hoon et al., 2010).
Lycopene	Tomato	Upregulation of caspase-3 and suppression of IL-6 (Minutoli et al., 2014)
Kolaviron	*Garcinia kola*	Inhibition of 5α-reductase and suppression of oxidative stress in the prostatic tissue (Kalu W. et al., 2016)
Secoisolariciresinol diglucoside	Flaxseed	Stimulation of increased expression of G-protein-coupled estrogen receptor 1 (Ren et al., 2016)

## Conclusion

While this review does not state emphatically that dietary polyphenols could replace the need for the existing therapies in the management of BPH, it suggests the promise some dietary polyphenols hold in BPH management which could be explored by researchers working in this field.

## Author Contributions

CE, KE, and WK reviewed the cited literature and wrote the manuscript. All authors read and approved the final manuscript.

## Conflict of Interest Statement

The authors declare that the research was conducted in the absence of any commercial or financial relationships that could be construed as a potential conflict of interest.
